# Computer aided greenness by design approach for resolving and quantifying Triamterene and Hydrochlorothiazide in pharmaceutical mixtures employing multiscale dynamics simulations

**DOI:** 10.1038/s41598-025-13486-2

**Published:** 2025-08-06

**Authors:** Ahmed Elsonbaty, Khaled Attala, Eman Darweish

**Affiliations:** Pharmaceutical Chemistry Department, Faculty of Pharmacy, Egyptian Russian University, Badr City, 11829 Cairo Egypt

**Keywords:** Greenness-by-Design, Mathematically manipulated spectral methods, Molecular and electronic dynamics simulations, Ratio-based UV spectrophotometry, Sustainability, Analytical chemistry, Quality control, Computational models

## Abstract

In this study, we present an established approach for the UV spectroscopic determination of Hydrochlorothiazide and Triamterene in pharmaceutical mixtures, leveraging the principles of Greenness-by-Design (GbD). By integrating molecular and electronic dynamics simulations, we have developed a greener analytical methodology that significantly reduces time and solvent waste. Our approach focuses on selecting a compromise solvent to minimize peak broadening, thereby enhancing spectral resolution and simplifying the analytical process. This work extends our ongoing efforts to establish the GbD concept in analytical chemistry, building on our previous research to promote sustainable and efficient analytical practices. The developed methods are based on simple mathematical manipulations, including Fourier self-deconvolution, absorption correction, and isoabsorptive point methods, along with ratio spectra approaches such as ratio difference and ratio derivative spectrophotometric methods. The Hydrochlorothiazide and Triamterene were determined with linear ranges of (1–18) µg/mL and (1–14) µg/mL, respectively. The detection and quantitation limits were in the range of (0.255–0.640) µg/mL, and (0.516–1.359) µg/mL, which reflects the method’s sensitivity. Moreover, a greenness assessment was performed to evaluate the sustainability of the proposed methods, indicating greener outcomes with a considerable reduction of the ecological footprint. This comprehensive approach underscores the potential of GbD in developing efficient, greener analytical methodologies for pharmaceutical analysis.

**Supplementary Information:**

The online version contains supplementary material available at 10.1038/s41598-025-13486-2.

## Introduction

The development of green analytical methodologies is an ongoing effort to achieve sustainability in chemistry research. Green analytical techniques are now essential not only for the protection of the environment but also for providing a safe research environment for human personnel^1^. The growing development of greenness assessment methods was noticed in the past decade with the reduction of the use of hazardous chemicals being the earliest step in the endeavor to establish green analytical methodologies^2,3^. Unfortunately, there is a modest effort exerted in developing approaches that merge the greenness concepts within the methodology development process^4^. Several obstacles hinder the integration of green chemistry concepts within analytical methodology development as limited choices and the effectiveness of green chemical reagents and solvents. This major defect can be bypassed by focusing efforts towards renovating methodology development approaches that reduce the overall experimentation required for optimizing the analytical process^5^.

Based on our previous research, the greenness by design (GbD) approach that aims to utilize both in-silico and in-vitro techniques in optimizing an analytical methodology has proven its efficiency in developing green UV spectrophotometric methodologies^6,7^. The GbD represents a significant leap towards achieving sustainability in analytical chemistry research. The GbD combines computational simulations with experimental work aiming to reduce the overall ecological footprint of the analytical process. This approach works by simulating the interaction between different solvents and solute molecules (analytes) to uncover the effects of solute-solvent interactions on the spectral interference among various solutes in a mixture. This is accomplished by examining the solute-solvent interactions at both the molecular and electronic levels to calculate several cheminformatics parameters that define how these interactions influence the peak broadening of the solute’s UV spectrum. This is achieved through the integration of molecular dynamics simulations (MD) and electronic dynamics (ED) via time-dependent density functional theory (TD-DFT) simulations, which together represent a powerful combination that provides extensive photochemical quantum data regarding the magnitude and nature of the solute-solvent interactions^8,9^. By analyzing these data we can compromise the solvent system used in the quantitation process one can choose the solvent with the minimal broadening effect on one or both solutes thus obtaining sharper UV spectral signals and reducing the overall commencing interference between solutes of the same pharmaceutical mixture^10,11^. This approach achieves a total reduction in the actual experimental work with reduced analytical effort attaining minimal ecological footprint of the developed methodology.

Thriving the pharmaceutical manufacture process of antihypertensive products is totally based on the development of quantitative analytical methodologies for multicomponent pharmaceutical products^12^. Hydrochlorothiazide (HCTZ) and Triamterene (TRIM) combination represent an example of a synergistic antihypertensive pharmaceutical mixture through which the patient benefits from the thiazide diuretic (HCTZ) action in lowering his blood pressure and overriding its side effects by another potassium-sparing diuretic (TRIM) which preserves patient’s potassium levels avoiding hypokalemia^13,14^. Reviewing the current literature reveals inadequate analytical techniques for the simultaneous quantitation of that pharmaceutical mixture as spectrophotometric^15–18^ and chromatographic methods^19–21^.

The reported UV spectrophotometric techniques were established on multivariate regression and wavelet transform which represent lengthy procedures, requiring sophisticated software to manage. Furthermore, the use of lengthy methodologies that require significant time investment may lead to decreased motivation among analysts^22,23^. This can result in a suboptimal allocation of cognitive resources and effort, potentially affecting the overall quality of the analysis^24^. At the same time, the reported chromatographic approaches suffered their inherent elevated organic solvent waste generation limiting their greenness merits^25–28^.

This study builds upon our ongoing research into GbD applications in UV spectroscopy, as outlined in our previous works^6,7^. In this context, we introduce a novel approach that utilizes TD-DFT-generated solute-solvent interaction energies coupled with MD simulations for solvent selection, rather than employing the full package of TD-DFT used in earlier studies to examine gap energies and solvent interactions with both excited and ground states. This streamlined implementation of a computer-aided GbD approach has enabled us to develop greener analytical procedures characterized by low solvent, computational time and energy consumption, thereby contributing to global sustainability goals in pharmaceutical analysis. The proposed methodologies were based on simple mathematically manipulated approaches for the spectral resolution of the investigated pharmaceutical mixture. Moreover, ratio spectra methodologies were also developed as universal UV spectrophotometric approaches to assess the sensitivity and resolution capabilities of the proposed mathematically manipulated methods.

However, it is important to acknowledge some potential limitations of the proposed UV methodologies. These include potential spectral interferences from co-existing substances in complex matrices, relatively lower sensitivity compared to hyphenated techniques (e.g., HPLC-MS), and limited selectivity in multi-component samples without appropriate wavelength selection or derivatization. Furthermore, while UV spectroscopy can effectively distinguish between closely related compounds, challenges may arise in achieving accurate results due to overlapping absorbance spectra. The GbD approach specifically addresses this challenge by optimizing solvent selection to achieve the lowest possible overlap, thereby enhancing the accuracy of the measurements.

Additionally, spectrophotometric methods may face difficulties with turbidity or colored excipients, which can affect absorbance readings. To address these concerns, we have briefly discussed possible strategies to mitigate these limitations, such as applying suitable sample preparation techniques and optimizing analytical parameters to improve reliability and accuracy.

Eventually, multiple greenness assessment protocols were applied to investigate the greenness outcomes of the developed GbD-based methodologies.

## Experimental

### Reagents and materials

EPICO, Egypt had generously given free samples of HCTZ and TRIM with proved purity of 99.65 and 98.97%, respectively. Maxzide 25 mg tablets were purchased online from Blink Health online pharmacy, claimed to contain 25 mg of HCTZ and 37.5 mg of TRIM. Ethanol spectroscopic grade was supplied by Honeywell, USA.

### Instrument and software

All UV spectrophotometric methodologies were conducted employing Jasco (V-750) UV spectrophotometer assisted by the Spectra Manager software (V.2, www.jascoinc.com/products/spectroscopy/molecular-spectroscopy-software). An Elma ultrasonic bath was utilized during the dissolution of standards and the ultrasonic assisted extraction from the investigated dosage form. A Centurion cool centrifuge was also utilized during the sample preparation steps. Molecular dynamic simulations were implemented by Molecular Operating Environment (MOE 2015, www.chemcomp.com/en/index.htm) with TD-DFT calculations performed by ORCA (V.4.2.1, orcaforum.kofo.mpg.de/app.php/portal) software utilizing an HP Zbook G3 workstation equipped with an Intel Core i7-6820HQ CPU running at 2.70 GHz^29,30^.

### Procedures

#### Standard stock and sample solutions preparation

Standard stock solutions of both HCTZ and TRIM were prepared by accurately weighing and transferring 10 mg of each drug standard to two separate 100 ml volumetric flask. Seventy milliliters of ethanol were added, and the mixture was shaken for 10 min vigorously and sonicated for another 15 min until complete dissolution was achieved attaining a homogenous clear solution. Finally, the volumes were adjusted using ethanol to obtain a standard solution of 100 µg/mL of each drug.

For sample preparation, ten tablets of Maxzide were finely ground, and an equivalent weight of one tablet was accurately measured and transferred to a 100 mL volumetric flask containing 70 mL of ethanol. The flask was sealed and shaken vigorously for 5 min, then subjected to ultrasonic dissolution for 20 min. After reaching room temperature, the volume was adjusted to the mark with ethanol.

A suitable aliquot was then transferred to a 50 mL Falcon tube for centrifugation at 10,000 rpm for 5 min. From the clear supernatant, 1 mL was withdrawn and quantitatively transferred into a 10 mL volumetric flask, where the volume was completed. Finally, an appropriate aliquot was taken from this flask and transferred to another 10 mL volumetric flask, with the volume completed using ethanol to achieve concentrations of 4 µg/mL for HCTZ and 6 µg/mL for TRIM. A suitable volume from this final solution was then transferred to a quartz cuvette for UV spectrophotometric scanning, using ethanol as the blank. Finally, the content of the tablet dosage form was assessed using the appropriate regression equation from each developed methodology.

#### Calibration curves construction

Calibration of the investigated drugs was achieved utilizing Jasco double beam spectrophotometer and all measurements were performed against ethanol as a blank.

#### Absorption correction method (ACM)

HCTZ and TRIM aliquots were properly obtained from their corresponding standard solutions, transferred into two sets of 10 mL volumetric flasks, and the remainder of the volume was adjusted with ethanol to generate concentration ranges of (4–18 µg/mL) and (2–14 µg/mL) for HCTZ and TRIM, respectively.

Each absorption spectrum was recorded versus ethanol as a baseline. The absorbance values of HCTZ at 271 nm (λ _max_) and TRIM at 271 and 361 nm were then plotted versus each relevant concentration in (µg/mL) for each component, and a correction factor (F_ac_) for TRIM at the corresponding wavelengths was derived to eliminate its interfering action with HCTZ at 271 nm.

#### Fourier self-deconvolution (FSD)

To prepare HCTZ and TRIM serial dilutions, the same technique outlined in standard stock solutions preparation was used. The zero-order absorption spectra of each component concentration vs. ethanol were recorded. Each recorded spectrum was deconvoluted using the FSD algorithm included in the spectra manager operating software with the Full width at half maximum (FWHM = 90). The amplitudes of HCTZ at 299 nm and TRIM at 366 nm were then plotted vs. each component’s concentration in (µg/mL).

#### Isoabsorptive point method (ISM)

Several aliquots of HCTZ and TRIM were accurately extracted from individual stock solutions and placed into a sequence of 10 mL volumetric flasks to produce different dilutions within the ranges of 1–18 µg/mL and 1–14 µg/mL, respectively. Each standard solution was analyzed separately with ethanol as a diluent, following which the absorbance levels for HCTZ and TRIM were recorded at 266.8 nm. A calibration was plotted for the concentration of HCTZ versus its corresponding absorbance at 266.8 nm then a regression equation was set up to predict the total concentration of both HCTZ and TRIM at the isoabsorptive point 266.8 nm in their prepared laboratory mixtures and pharmaceutical dosage forms. Finally, the pure concentration of TRIM was determined at the zero order spectrum at the no contribution point 361 nm.

### Ratio spectra spectrophotometric methods

#### Ratio difference spectrophotometric method (RDF)

Standard solutions of HCTZ and TRIM with concentrations (1–18 µg/mL) and (1–14 µg/mL), respectively, were prepared following the procedure outlined under stock solution preparation. The absorption spectrum for each drug solution was recorded against ethanol as a blank. Subsequently, the spectrum of HCTZ was divided by the spectrum of TRIM (10 µg/mL) as the divisor, while the spectrum of TRIM was divided by the spectrum of HCTZ (4 µg/mL) as the divisor. The differences in amplitudes of the resulting ratio spectra were calculated at ΔP (273–293 nm) for HCTZ and ΔP (244–274 nm) for TRIM. These differences were then plotted against the corresponding concentrations (µg/mL) for each component.

#### Ratio derivative spectrophotometric method (RDM)

The procedure described in standard stock solutions preparation was utilized to prepare serial dilutions of HCTZ and TRIM across a concentration range of (1–18 µg/mL) and (1–14 µg/mL) for both compounds. Subsequently, the zero-order absorption spectra for HCTZ and TRIM were obtained. These spectra were then divided by the spectra of TRIM and HCTZ by their respective concentrations of 4 & 10 µg/mL, respectively, thus serving as the divisors. The resulting ratio spectra were analyzed to compute the first derivative, and the corresponding amplitudes at 283 nm and 251 nm were recorded for the quantification of HCTZ and TRIM, respectively.

### Molecular and electronic dynamics & photochemical quantum investigations

MD simulations were performed utilizing molecular operating environment 2015 (MOE) based on the Amber: EHT10 force-field^31^ to investigate the behavior of both HCTZ and TRIM in different solvent environment (water, methanol, acetonitrile and ethanol). Various descriptors as solvation energy, hydrogen bonding and non-bonding interaction energies were calculated for both molecules in each solvated environment. The TD-DFT calculations were implemented based on the orca software utilizing the B3LYP hybrid functional assisted by the RIJCOSX functional^32^ and the polarized triple-zeta basis set (def2-TZVP). The conductor-like polarizable continuum model (CPCM) solvation model was utilized to simulate the effect of different investigated solvents. After normal convergence of the preceded calculations, the solute-solvent interaction energies were calculated.

## Results and discussion

The application of Green by Design (GbD) in UV spectroscopy has proven effective in developing environmentally friendly spectrophotometric methods^6,7^. In this study, we conducted extensive molecular mechanical, photochemical quantum and electronic dynamics investigations on a binary mixture of HCTZ and TRIM to explore how different solvents affect the interference between the UV spectral signals of these drug molecules.

Using MD simulations, we calculated several cheminformatic descriptors, including hydrogen bonding capacity, solvation energy, and non-bonded interactions within each solvent environment. Additionally, TD-DFT calculations were performed to examine solute-solvent interactions and the corresponding dipole moments for each drug molecule in various solvents. The data obtained from both MD and TD-DFT simulations were helpful in optimizing the solvent system for developing UV spectroscopic methodologies^33^. Our goal was to minimize spectral interference between the two drugs and reduce the analytical effort required to differentiate their signals in combined dosage forms.

**Fig. 1:**
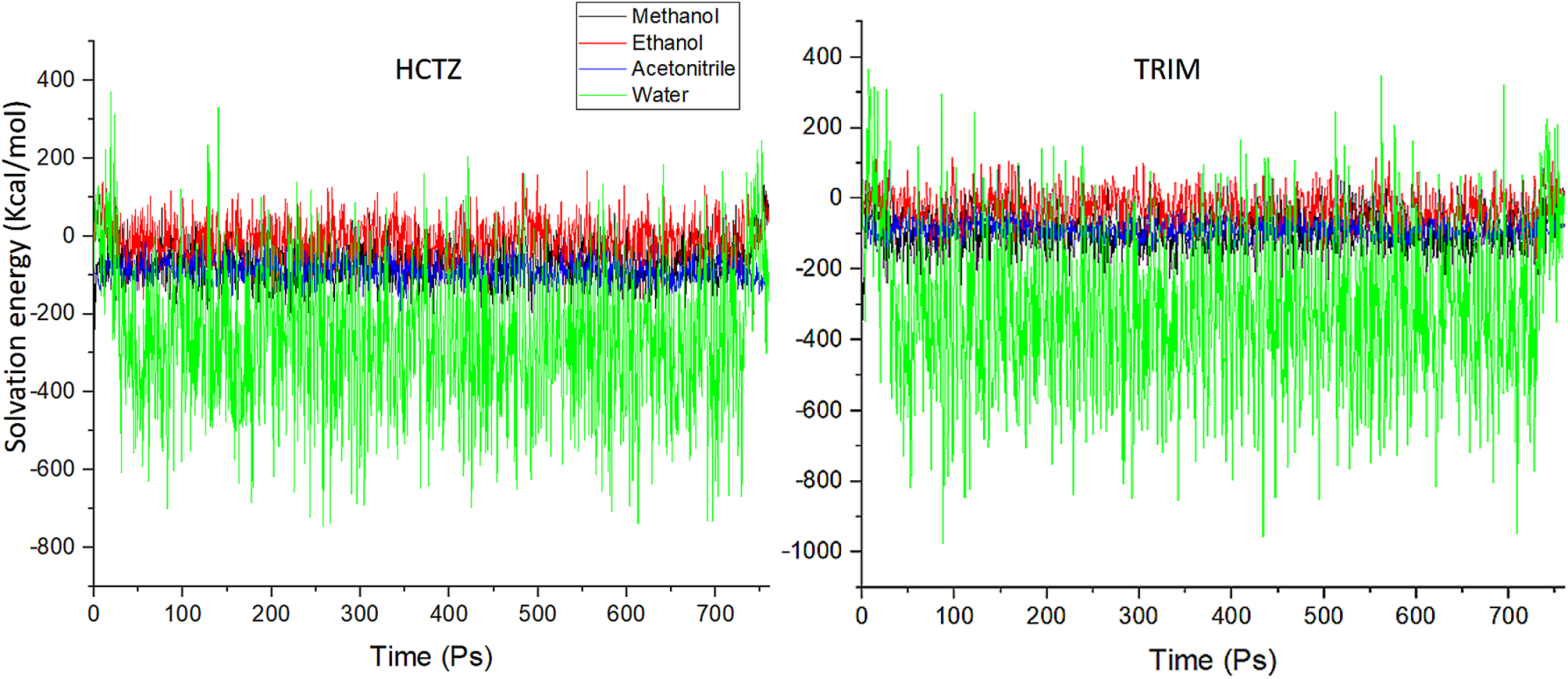
Solvation energy profile for both HCTZ and TRIM in water, ethanol, methanol and acetonitrile.

Building on this foundation, our current work implements the GbD approach to identify a suitable solvent that achieves optimal spectral resolution for the pharmaceutical mixture under study (FigureS1). Traditional laboratory testing of various solvents often results in significant waste; however, the in-vitro GbD approach minimizes waste by confining it to the application stage. Furthermore, this method reduces analysis time and effort by enhancing spectral resolution, thereby decreasing the analytical workload compared to conventional UV spectroscopic methods.

When compared to other analytical approaches^17,34^ such as classical solvent screening and trial-and-error methods, the GbD approach not only streamlines the process but also enhances efficiency and sustainability in green analytical chemistry. This comparative advantage highlights the potential of GbD as a more effective alternative for solvent selection in spectroscopic applications.

### Molecular dynamics simulation

**Fig. 2:**
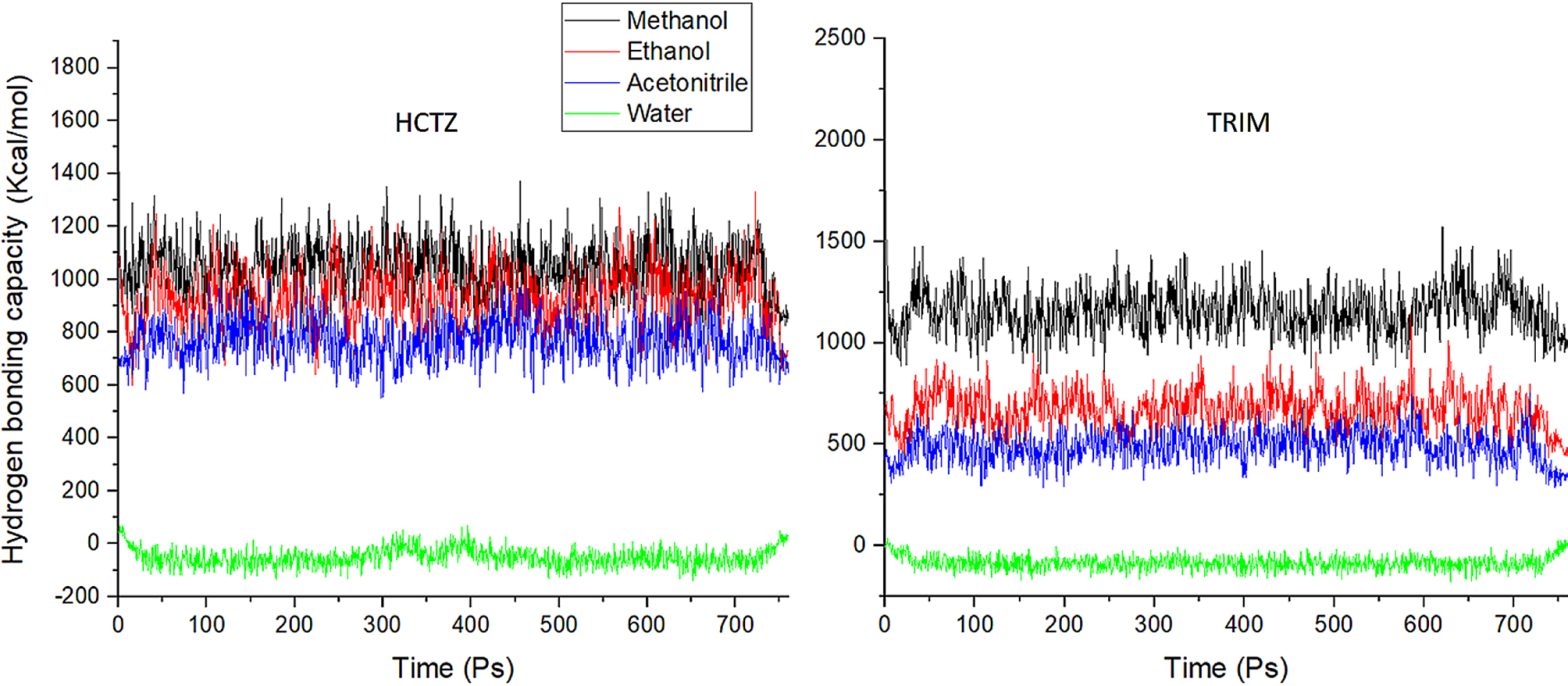
Hydrogen bonding capacity for both HCTZ and TRIM in water, ethanol, methanol and acetonitrile.

After the 760 Ps simulation, the trajectory files for each drug in each solvated environment was analyzed regarding solvation, interaction energy, and hydrogen bonding capacity. The analysis of solvation energies revealed an endothermic behavior of both HCTZ and TRIM in methanol, ethanol and acetonitrile, while showing an exothermic dissolution pattern in water as in Fig. 1. The hydrogen bonding (HB) capacities of HCTZ and TRIM in the investigated solvents were found to correlate with their respective solvation profiles. Both molecules exhibited higher HB capacities in methanol and ethanol, while showing significantly lower capacities in acetonitrile, with the lowest observed in a water environment, as illustrated in Fig. 2.

Methanol and ethanol effectively interacted with both HCTZ and TRIM, facilitating the establishment of hydrogen bonds. In contrast, acetonitrile, being an aprotic solvent, demonstrated lower HB capacities for both molecules compared to methanol and ethanol.

In the case of water, we propose that the observed lowest HB capacities can be attributed to the unique characteristics of water as a solvent. Water possesses a robust intramolecular hydrogen bonding network that extensively solvates solute molecules. This extensive solvation can inhibit effective solute-solvent hydrogen bond formation due to steric hindrance and competition from surrounding solvent molecules. Thus, the strong hydrogen bonding interactions within the water network may limit the availability of hydrogen bond sites for solute interactions, further explaining the reduced HB capacities observed in this solvent^35^.

**Fig. 3:**
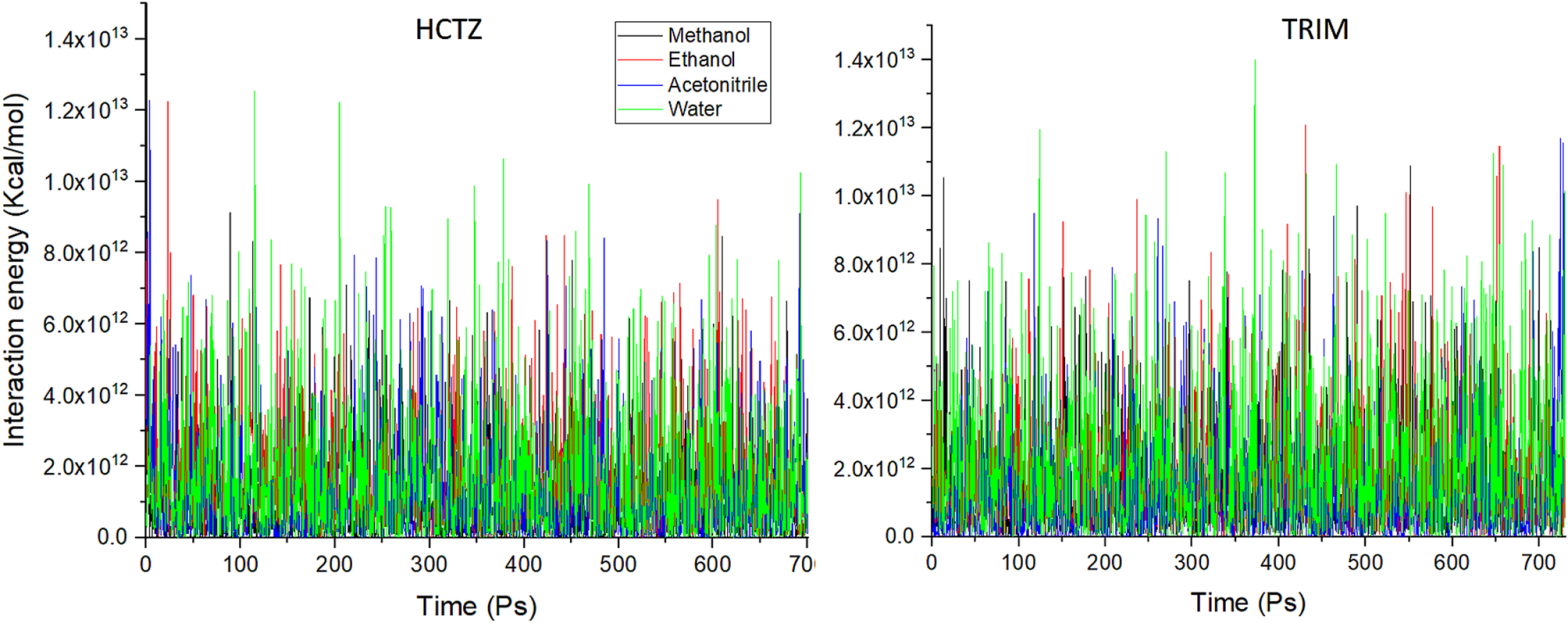
Interaction energy plot for both HCTZ and TRIM in water, ethanol, methanol and acetonitrile.

The analysis of the interaction energies profile for the two molecules in the four solvents was very complex due to interfering interaction profiles for both drugs in the investigated solvents as in Fig. 3.

The initial analysis of the data obtained from MD simulations provided compelling evidence of significant interactions between the water solvent and both HCTZ and TRIM. These findings prompted us to initially exclude water from further consideration, given the exothermic dissolution behavior of both compounds in aqueous environments^36^. The interactions between solute and solvent are critical factors influencing the resolution of UV spectra in their pharmaceutical mixtures. Understanding the nature and magnitude of these interactions necessitates further investigation at the sub-molecular level to elucidate their extent and implications for the analytical efforts required to resolve the investigated mixture.

### Electronic dynamics and photochemical quantum investigations

In this study, we utilized the B3LYP hybrid functional, supported by the RIJCOSX functional and the def2-TZVP basis set, to investigate solute-solvent interaction energies critical to our GbD methodology in UV spectroscopy. The B3LYP functional is recognized for its balance of computational efficiency and accuracy in predicting electronic properties, effectively capturing the physics of excited states. The RIJCOSX functional enhances computational efficiency by reducing the scaling of calculations, allowing for faster evaluations of electron repulsion integrals, particularly beneficial for larger systems with multiple solvent interactions^32^. The def2-TZVP basis set provides an optimal balance between computational demand and detail, enabling accurate electronic structure calculations and improved representation of electron distribution in excited states. Together, these parameters create a robust framework for our analysis^37^.

The TD-DFT calculations yielded comprehensive data regarding the electronic configurations and excited states of the investigated compounds. Furthermore, these calculations provided valuable insights into the energy characteristics of the solvated systems and variations in dipole moments of each compound within different solvent environments. These parameters were crucial for establishing a relationship between the solvent systems employed and their effects on the spectral broadening of the investigated compounds.

**Fig. 4:**
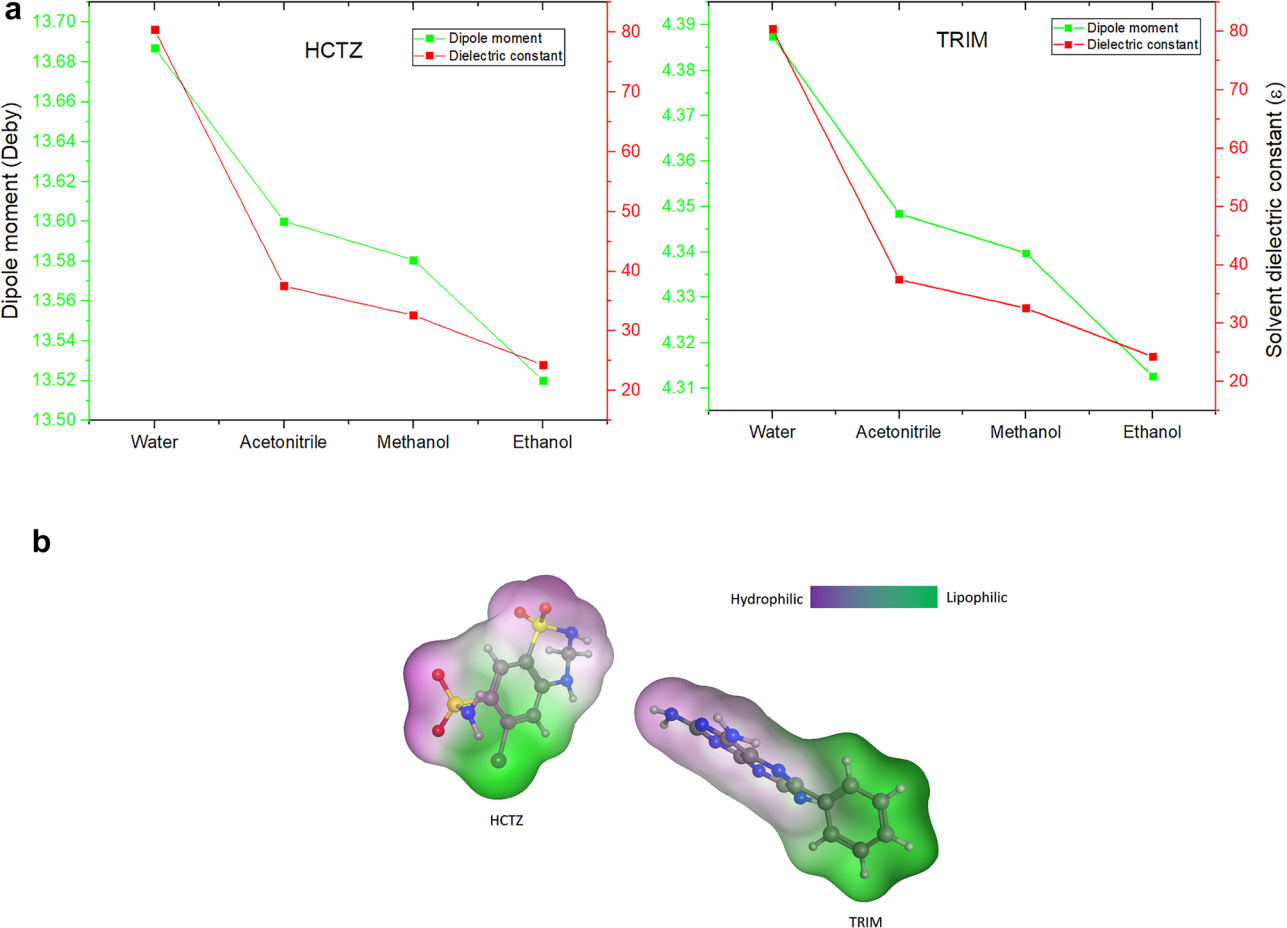
**(a)** Molecular dipole moment for both HCTZ and TRIM in different solvents versus solvent dielectric constant. **(b)** Hydrophobic mapping for both HCTZ and TRIM showing lipophilic/hydrophilic molecular surface area. This figure was created by the authors using the Molecular Operating Environment (MOE 2015) and its molecular surface lipophilicity mapping tool. For more information, please visit https://www.chemcomp.com.

The observed consistency between the calculated molecular dipole moments and the polarity of the solvents, as indicated by the dielectric constant (ɛ), demonstrated the influence of solvent-induced polarization on the solute^38^. This alignment further validates the accuracy of the DFT calculations, as illustrated in Fig. 4a.

Additionally, the investigation of the molecular dipole moment provided valuable insights into the molecular polarity and the influence of various solvents on the investigated compounds. TRIM was found to be less polar than HCTZ, with dipole moment values ranging from 4.313 to 4.388 Debye. In contrast, HCTZ exhibited dipole moment values ranging from 13.520 to 13.687 Debye, indicating a significantly higher polarity. Moreover, the hydrophobicity mapping of both molecules revealed a larger hydrophobic center on TRIM in comparison with HCTZ as shown in Fig. 4b**.** These observations suggest that different solute-solvent interaction behaviors are expected within the solvated molecular systems of both compounds.

Given that HCTZ is the more polar compound, it is anticipated to be particularly sensitive to solvent interactions, especially with highly polar solvents such as water. These interactions may result in notable changes in its UV spectrum, manifesting as shifts in peak position and broadening of absorption peaks.

**Fig. 5:**
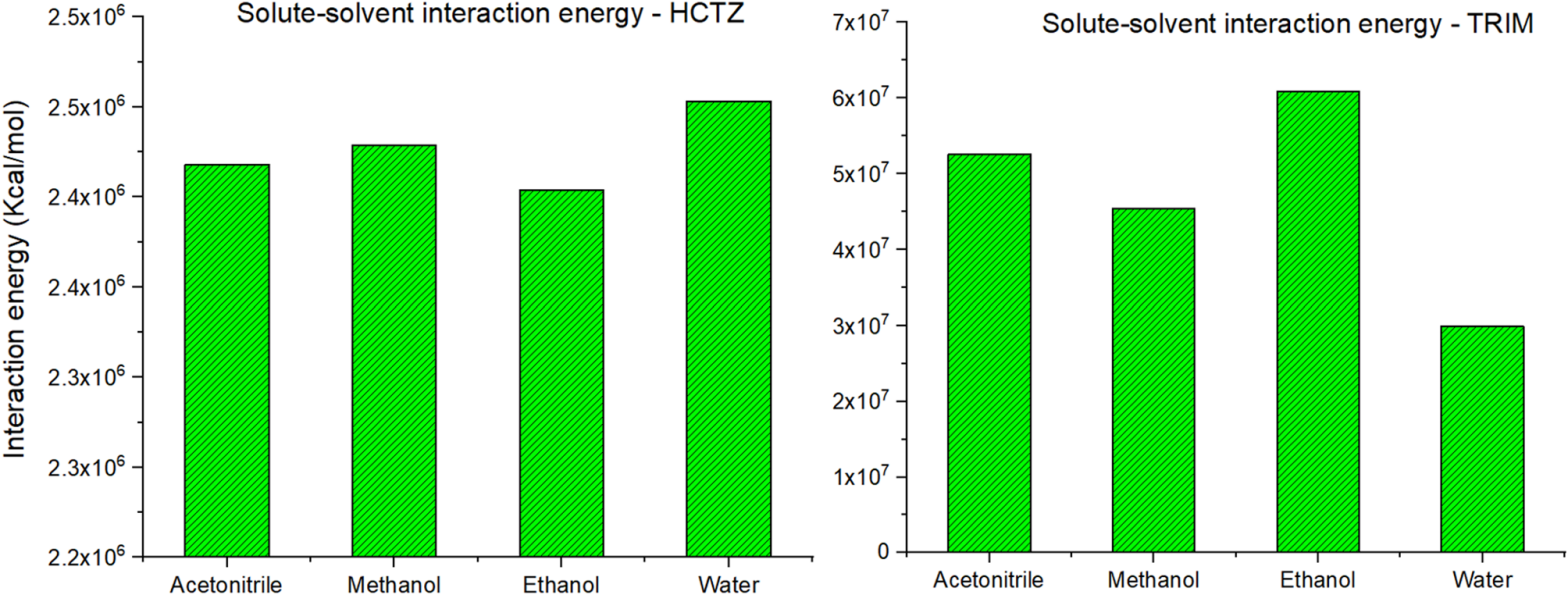
Solute-solvent interaction energies for HCTZ and TRIM in different solvents calculated by TD-DFT simulation.

The interaction energies between the outermost valence shell electrons of TRIM and HCTZ with their surrounding solvent molecules were also investigated and are shown in Fig. 5. HCTZ exhibited the highest interaction energies with water, consistent with its exothermic behavior in an aqueous environment as observed from MD simulations. This finding led to the exclusion of water as a potential solvent for UV spectroscopic analysis, as the higher solvent interactions with the molecule, the greater broadening of its spectral peaks was observed, increasing the chance of interference with other molecular signals in a pharmaceutical mixture. Conversely, HCTZ showed the lowest interactions with ethanol making it a potential candidate for UV spectral measurements.

For TRIM, water was the least interactive solvent, followed by methanol, acetonitrile, and finally ethanol, as shown in Fig. 5. Although water showed the least interaction energy with TRIM it showed an exothermic pattern of dissolution in reverse to expectations as seen in Fig. 1. This anomaly can be illustrated by revising the structural properties of TRIM, due to its elevated hydrophobicity it causes disruption of the hydrogen bond network between water molecules forcing them to reorganize in more stable molecular arrangement around the hydrophobic regions of the solute^39^ which eventually result in energy release to the system so that the dissolution profile seems to be exothermic despite the lower solute-solvent interactions.

The rationale behind solvent selection is well supported by both MD and TD-DFT data. While our findings indicate that ethanol minimally interacts with HCTZ and significantly interacts with TRIM, it is important to note that the mixture of HCTZ and TRIM exhibits a one-way interaction. Specifically, the main peak of TRIM, when measured in ethanol, aligns at 361 nm, which coincides with a region of minimal contribution from HCTZ due to the solvent’s narrowing effect on HCTZ’s peaks at 271 nm and 221 nm.

As illustrated in Figure S2a, the comparison of interaction energies reveals a significantly stronger interaction between ethanol and TRIM compared to HCTZ, supporting the notion of ethanol’s selective solvation properties. This enhanced interaction with TRIM is crucial for its solubility and stability in solution. Furthermore, Figure S2b shows the full range overlay spectra of both HCTZ and TRIM in ethanol, highlighting how ethanol broadens the TRIM peaks, particularly its n-π* transition at 361 nm, while exerting a less significant broadening effect on the HCTZ peaks. This characteristic simplifies the analytical task of resolving the two compounds, especially when employing simple straightforward methodologies such as the ACM, FSD and ISM methods.

Thus, despite the differing interaction strengths, the solvent ethanol facilitates effective separation and analysis of HCTZ and TRIM, reinforcing its selection as the optimal solvent for our study.

### Spectrophotometric approaches

#### Absorption correction method (ACM)

In ACM, a simple mathematical technique was utilized to analyze the binary mixture of HCTZ and TRIM by focusing on the extended regions of their overlapping spectra^40^. The concentration of TRIM was effectively determined using its zero-contributing segment at 361 nm within the overlapping spectra. To mitigate interference at the λ _max_ of HCTZ, an interference-correcting factor (F_ac_) was established for TRIM. This factor was derived by dividing the absorbance of TRIM at 271 nm by its absorbance at 361 nm.

**Fig. 6:**
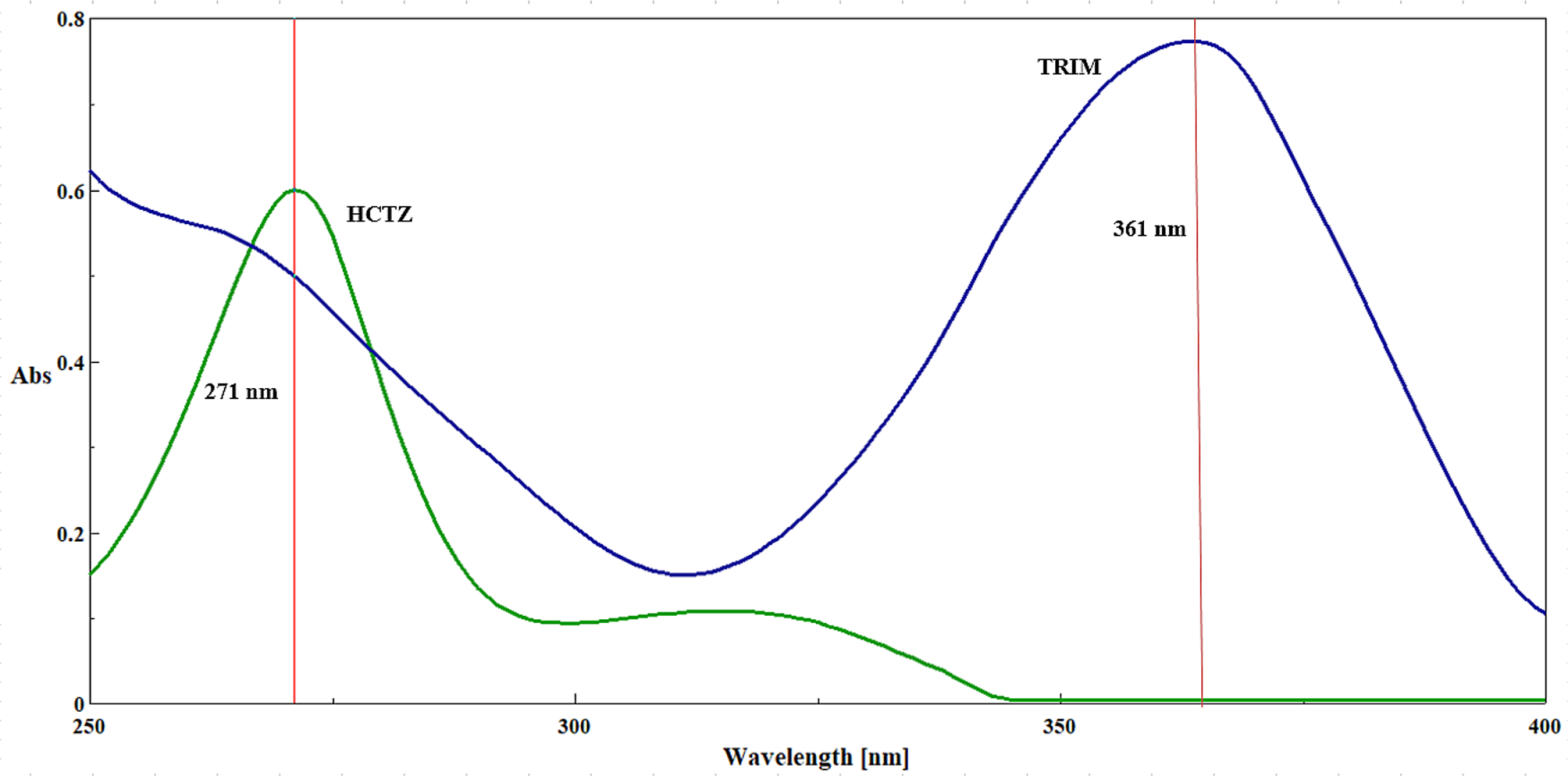
Zero-order UV overlain spectra of both HCTZ and TRIM, each at a concentration of 10 µg/mL, illustrating the determination of both drugs at 271 and 361 nm, respectively by the absorption correction method.

A calibration curve for TRIM was created at 361 nm across a concentration range of 2–14 µg/mL (Fig. 6), and the associated regression equation, detailed in Table 1, was used to ascertain its concentration in both laboratory-prepared mixtures (Table 2) and pharmaceutical samples (Table 3). Importantly, the absorbance measured at 271 nm, which aligns with the λ _max_ of HCTZ, does not provide an accurate representation of pure HCTZ concentration due to ongoing interference from TRIM at this wavelength (Fig. 6).

**Table 1:** Regression and validation data for the determination of HCTZ and TRIM by the proposed spectrophotometric methods.

Parameters	HCTZ					TRIM				
	FSD	ISM	ACM	RDF	RDM	FSD	ISM	ACM	RDF	RDM
Wavelength (nm)^a^	299	266.8	271	Δ 273–293	283	366	361	361	Δ 244–274	251
Calibration range (µg/ml)	1–18	2–16	4–18	1–18	1–18	2–12	2–14	2–14	1–14	1–14
Slope	0.0879	0.0569	0.0690	0.0860	0.0062	0.1768	0.0803	0.0803	1.007	0.065395
Intercept	0.0184	0.0031	−0.0517	0.0083	0.0010	−0.0573	0.0049	0.0051	−0.0550	−0.02194
r	0.9998	0.9995	0.9992	0.9999	0.9997	0.9996	0.9993	0.9995	0.9997	0.9996
LOD (µg/mL)	0.255	0.378	0.640	0.170	0.319	0.449	0.352	0.352	0.283	0.318
LOQ (µg/mL)	0.774	1.147	1.939	0.516	0.964	1.359	1.068	1.068	0.857	0.962
Accuracy ^b^ (% RSD)	99.12	99.58	100.11	99.97	99.96	100.41	99.01	99.85	100.73	100.26
	0.521	0.556	0.782	0.144	0.544	0.631	0.801	0.675	0.685	1.72
Precision (% RSD) ^c^ - Repeatability - Intermediate precision	0.452 0.852	0.585 0.732	0.771 1.011	0.189 0.326	1.67 1.86	0.784 0.986	0.544 1.241	0.673 1.854	0.659 0.731	2.434 2.479

^a^ The peak amplitude of measurement of each proposed method.

^b^ Average of nine determinations (three concentrations repeated three times).

^c^ % RSD of nine determinations (three concentrations repeated three times).

**Table 2:** Determination of HCTZ and TRIM in their laboratory prepared mixtures by the proposed UV spectrophotometric methods.

HCTZ	TRIM	% Recovery ^a^									
conc. (µg/ml)		HCTZ						TRIM			
		FSD	ISM	ACM	RDF	RDM	FSD	ISM	ACM	RDF	RDM
4	6	99.96	98.05	99.19	99.69	100.56	100.75	99.66	99.66	99.88	98.62
6	6	99.97	99.43	99.24	100.78	99.65	100.18	98.39	98.39	98.01	98.87
4	12	99.58	99.08	100.62	98.52	100.97	101.85	100.47	100.47	100.74	100.41
4	8	99.47	100.29	99.60	101.72	98.14	100.90	100.05	100.05	98.71	99.01
8	6	98.69	98.06	99.15	100.16	101.02	99.86	99.52	99.52	99.43	99.38
Mean ± SD		99.53 ± 0.522	98.98 ± 0.951	99.56 ± 0.618	100.17 ± 1.19	100.07 ± 1.21	100.71 ± 0.767	99.62 ± 0.778	99.62 ± 0.778	99.35 ± 1.05	99.26± 0.627

^a^ average of three determinations.

**Table 3:** Determination of HCTZ and TRIM in the prepared dosage form by the proposed spectrophotometric and reported methods.

Parameters	Proposed methods											
	HCTZ					TRIM					Reported methods	
	FSD	ISM	ACM	RDF	RDM	FSD	ISM	ACM	RDF	RDM	HCTZ	TRIM
Number of measurements	3	3	3	3	3	3	3	3	3	3	3	3
Mean % recovery ^a^	99.57	99.46	99.86	100.23	100.89	100.59	99.64	99.64	100.09	100.87	101.22	101.65
% RSD	1.138	1.629	1.600	1.54	1.24	0.339	1.317	1.317	0.78	0.87	0.634	1.044
Variance	1.295	2.655	2.560	2.372	1.54	0.038	1.755	1.755	0.608	0.757	0.402	1.090
Student’s t-test ^b^	2.20	1.75	1.37	0.830	0.308	1.68	2.08	2.08	2. 07	0.994	- (2.78)	- (2.78)
F-value ^b^	0.31	0.15	0.16	1.245	1.24	0.11	0.63	0.63	1.79	1.44	- (19)	- (19)

^a^ average of three determinations.

^b^ The values in parenthesis are tabulated values of “t” and “F” at confidence interval 95% (*P* = 0.05).

To determine the concentration of HCTZ, the absorbance of TRIM at 361 nm, adjusted by the F_ac_, must be subtracted from the absorbance of HCTZ at 271 nm across a concentration range of 4–18 µg/mL. This relationship can be expressed as ΔP (271 nm _HCTZ_ – (361 nm _TRIM_ × F_ac_)).

#### Fourier self-deconvolution (FSD)

FSD is a computational technique utilized to resolve overlapping spectral peaks by effectively narrowing their bandwidths, thereby facilitating the differentiation of individual peaks. As proposed by Kauppinen^34^FSD addresses the instrumental distortions inherent in spectrophotometric measurements, defined as physical convolution, where the measured spectrum M(v) can be expressed as:

1 $$M{\text{ }}\left( v \right){\text{ }} = {\text{ }}E{\text{ }}\left( v \right){\text{ }}*{\text{ }}G{\text{ }}\left( v \right)$$

Here, G (v) represents the instrumental distortion function, and * denotes the convolution integral. FSD reverses this convolution process, allowing for the extraction of the original spectrum E (v) through the inverse Fourier transformation:

2 $$IE(x) = \frac{1}{{{F^{ - 1}}\;(G\;(v))}}IM(x)$$

The final form of E (v) can be derived as:

3 $$E (v) =\:\:F\left\{\frac{D\:(L,\:x)}{\text{exp}\left\{\pi\:\sigma\:\left|x\right|\right\}}\right\}*M\:\left(v\right)$$

In this context, an apodization function D (L, x) is employed to mitigate the effects of noise, which can significantly impact spectral clarity. The trade-off between resolution and signal intensity is quantitatively assessed through the signal-to-noise ratio (SNR). FSD is used in UV spectroscopy to enhance spectral resolution by making overlapping peaks more distinguishable along the wavelength (X) axis. This technique works by mathematically reducing peak broadening, which helps reveal individual components in complex spectra. However, this improvement in resolution can come at the cost of intensity (Y-axis) information, as FSD may amplify noise especially in low-signal regions.

While this doesn’t always lead to a higher SNR in the conventional sense, it often improves the apparent SNR by making the peaks easier to differentiate. As a result, FSD enables more accurate identification and semi-quantitative analysis of overlapping signals when applied carefully^41–43^.

In our study, we leveraged the GbD approach to simultaneously determine both HCTZ and TRIM in their binary mixture. A significant limitation of FSD is its inability to distinguish between components in a binary mixture due to extensive overlap in their spectral signals. However, in our specific mixture, TRIM was prominently situated in the zero-contribution zone of the overlapped spectra, facilitating its resolution from HCTZ. Conversely, resolving HCTZ from TRIM posed a challenge due to considerable overlap around the λ _max_ of HCTZ. The GbD approach was influential in this context, allowing us to select an appropriate solvent that minimizes spectral interference and maintains narrow spectral bandwidths for both compounds. This reduction in overlap significantly aided FSD in the simultaneous resolution of both compounds in their binary pharmaceutical mixture^44^.

**Fig. 7:**
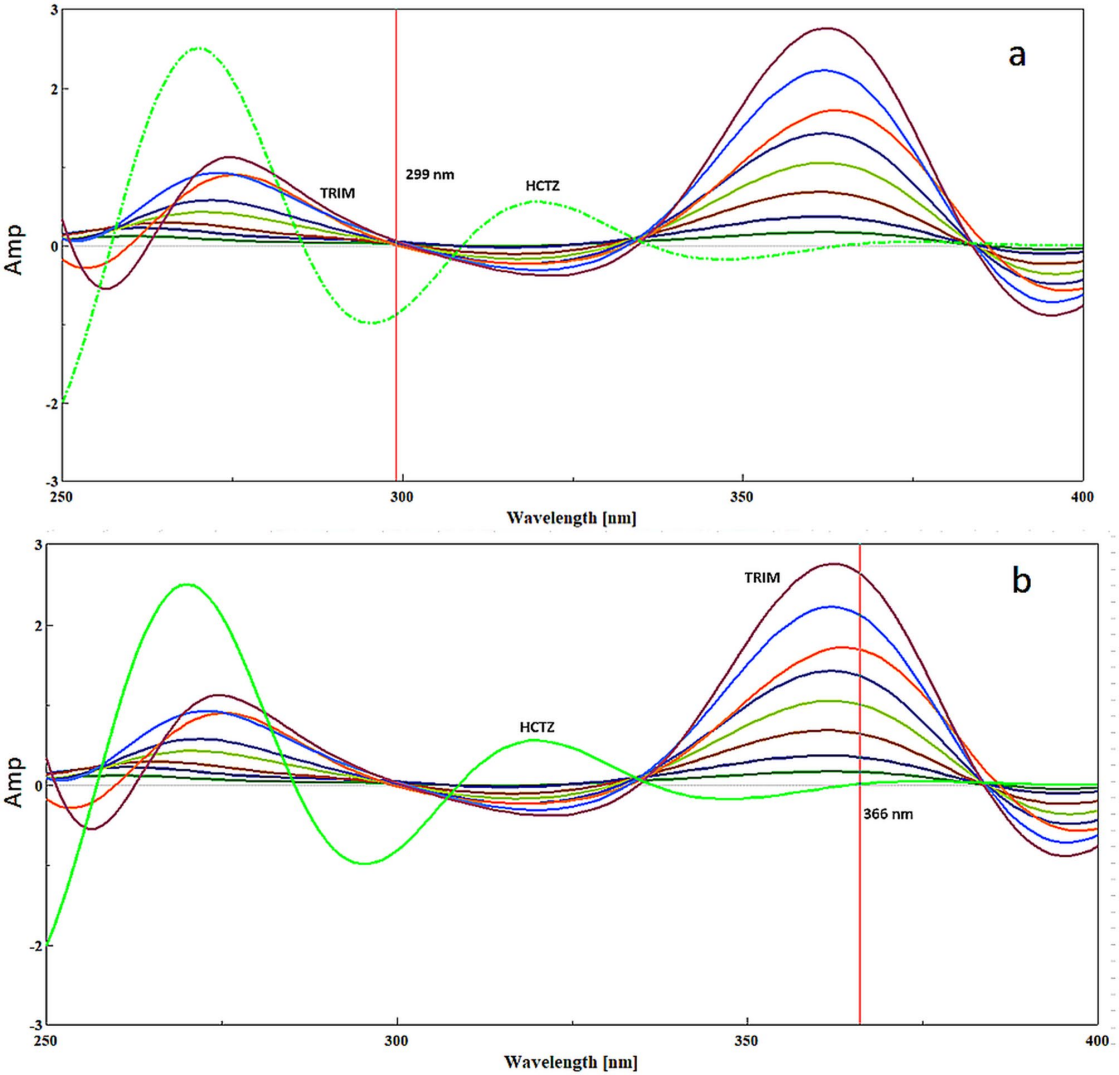
**(a)** Deconvoluted overlain UV spectra of both HCTZ and TRIM for the determination of HCTZ at 299 nm by Fourier self-deconvolution method. **(b)** Deconvoluted overlain UV spectra of both HCTZ and TRIM for the determination of TRIM at 366 nm by Fourier self-deconvolution method.

By applying the FSD method, we successfully resolved both HCTZ and TRIM. The zero-order spectra of HCTZ and TRIM were recorded and subsequently deconvoluted as outlined previously. A calibration curve was then constructed by plotting the amplitudes of the deconvoluted spectra of HCTZ and TRIM at 299 nm and 366 nm, respectively, against their corresponding concentrations (Fig. 7a&b). Each regression equation was utilized to determine the concentrations of HCTZ and TRIM in both laboratory-prepared mixtures (Table 2) and formulated dosage forms (Table 3).

#### Isoabsorptive point method (ISM)

**Fig. 8:**
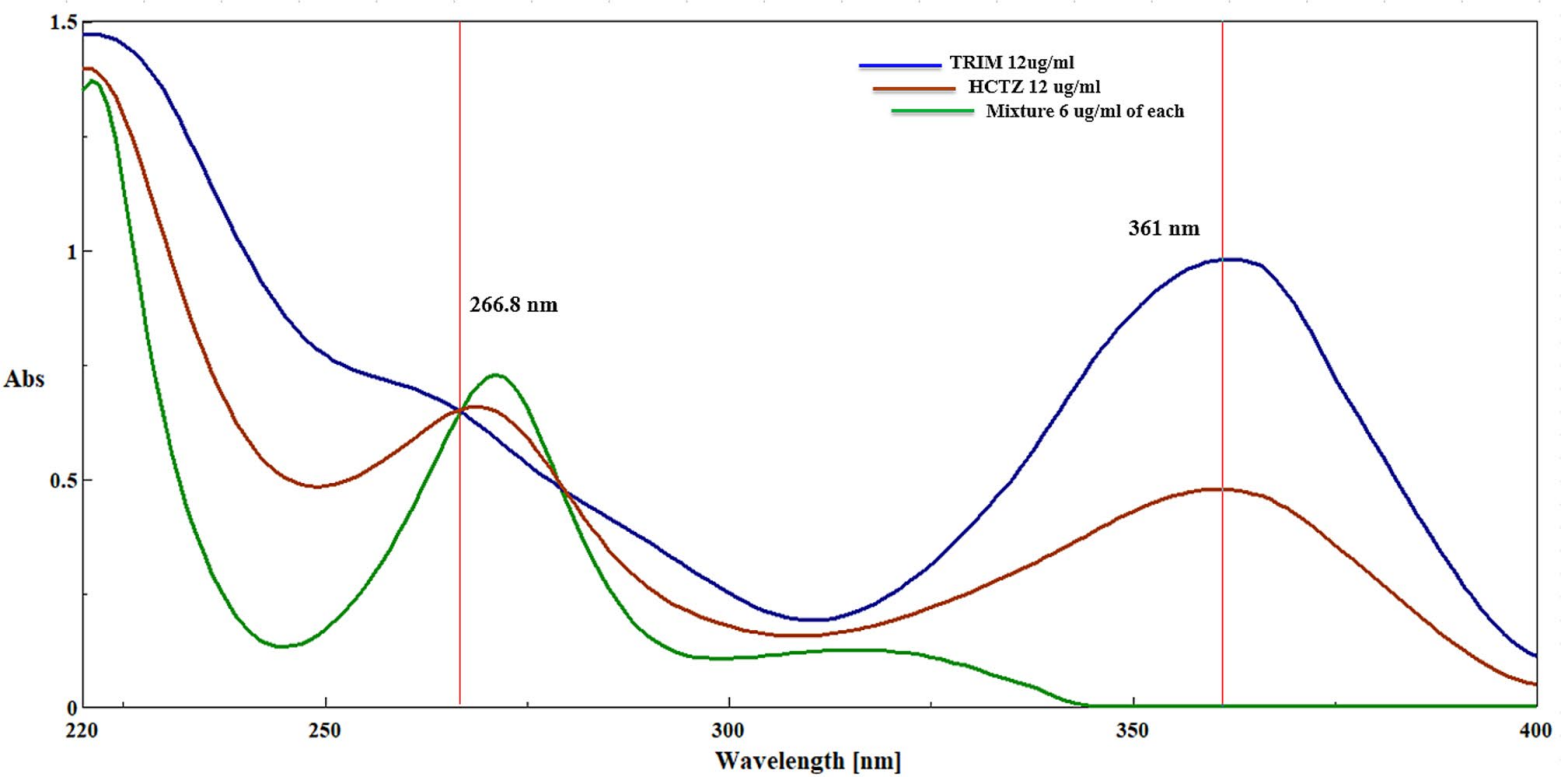
UV overlain spectra of HCTZ and TRIM (12 µg/mL) with their synthetic mixture (6 µg/mL of each) showing their isoabsorptive point at 266.8 nm.

The ISM is a straightforward mathematical approach used to resolve spectral interference in a binary mixture, particularly when one component can be determined at its λ _max_ with zero contribution from the second component^45^. This method relies on the isoabsorptive point, where both components contribute equally to the overall absorbance. In our study, HCTZ and TRIM exhibited an isoabsorptive point at 266.8 nm (Fig. 8). A calibration curve for HCTZ was established over the concentration range of 1–18 µg/mL, and the resulting regression equation was employed to determine the total concentrations of both TRIM and HCTZ in pharmaceutical dosage forms and laboratory-prepared mixtures.

TRIM was easily quantified at 361 nm without interference from HCTZ. Consequently, the pure concentration of HCTZ in the dosage form was calculated by subtracting the concentration of TRIM from the total concentration determined at 266.8 nm. ISM is highly selective when one component can be measured at its λ _max_ with no contribution from the other component (HCTZ). This makes it ideal for scenarios where such conditions are met. The method is sensitive within the defined concentration range (2–16 µg/mL for HCTZ and 2–14 µg/mL for TRIM), allowing for precise quantification. ISM typically requires fewer manipulations, contributing to a more environmentally friendly analytical process.

#### Ratio difference spectrophotometric method (RDF)

Achieving successful spectrophotometric analyses necessitates careful selection of divisor concentrations and appropriate wavelength pairs^46,47^. A range of concentrations for each divisor was assessed to determine the most effective parameters. For quantifying HCTZ, TRIM at 10 µg/mL was employed as a divisor to minimize interference, while HCTZ at 4 µg/mL was chosen as the divisor for TRIM. This selection was based on criteria such as repeatability, signal-to-noise ratio, recovery percentage, and the linearity of the resulting ratio spectra observed during preliminary trials.

**Fig. 9:**
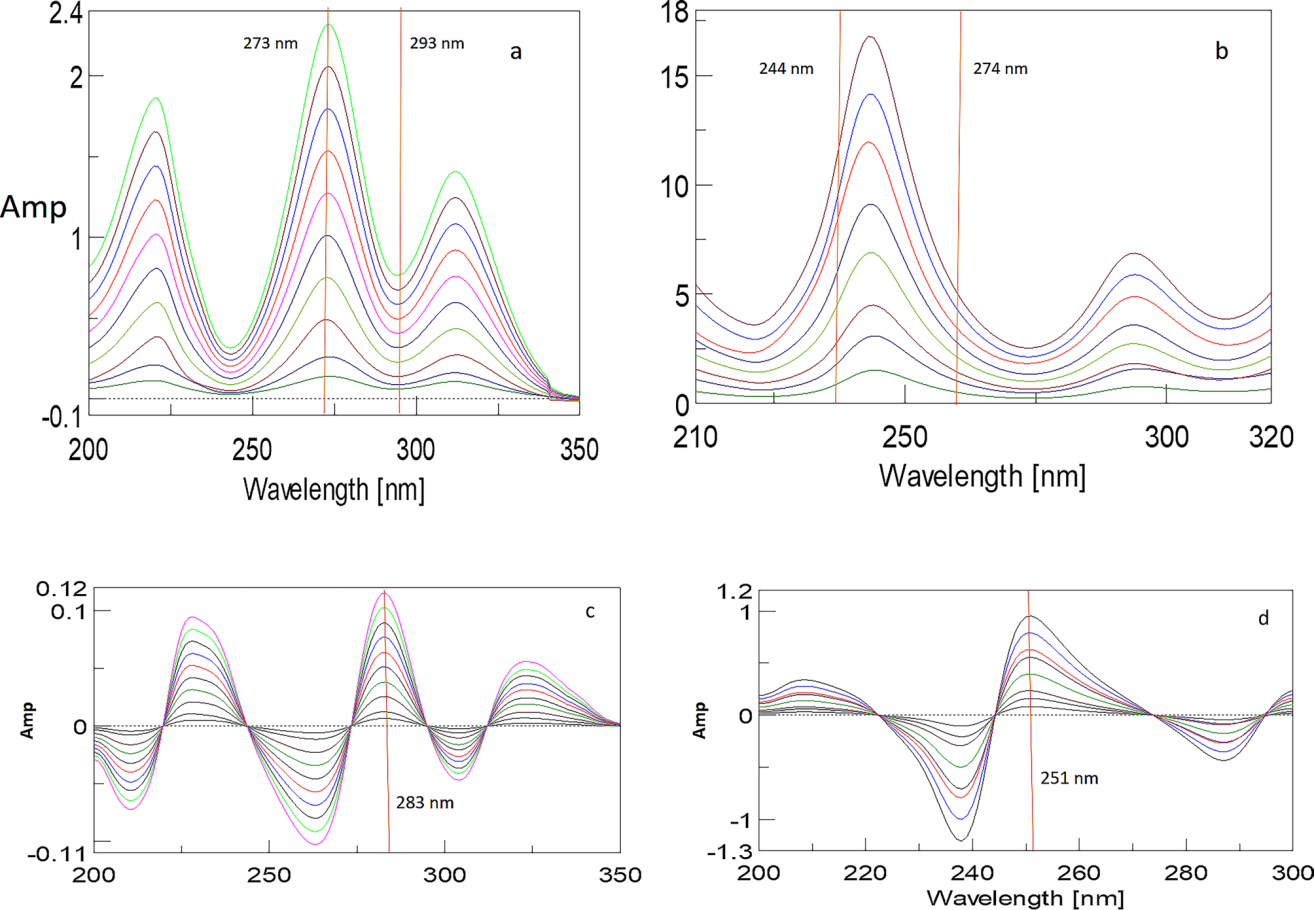
**(a)** Ratio spectrum of HCTZ using 10 µg/mL TRIM as a devisor showing difference points at 273 and 293 nm for the determination of HCTZ by the ratio difference spectrophotometric method. (**b)** Ratio spectrum of TRIM using 4 µg/mL HCTZ as a devisor showing difference points at 244 and 274 nm for the determination of TRIM by the ratio difference spectrophotometric method. **(c)** First derivative of the ratio spectrum of HCTZ using 10 µg/mL TRIM as a devisor for the determination of HCTZ at 283 nm by the ratio derivative spectrophotometric method. (d**)** First derivative of the ratio spectrum of TRIM using 4 µg/mL HCTZ as a devisor for the determination of TRIM at 251 nm by the ratio derivative spectrophotometric method.

It is essential that the selected wavelength pairs exhibit clear peak amplitudes in their ratio spectra. Satisfactory linearity was confirmed at each wavelength independently. Several wavelength pairs were tested for their quantification effectiveness, with (ΔP273–293 nm) and (ΔP244–274 nm) identified as the most suitable for determining HCTZ (Fig. 9a) and TRIM (Fig. 9b), respectively. The absolute amplitude differences at these wavelength pairs were found to be directly proportional to the concentrations of the analytes studied (Table 1).

Ratio spectra based methodologies can achieve selectivity by carefully choosing divisor concentrations and wavelength pairs that minimize interference. The sensitivity of RDF is contingent upon the choice of divisor concentrations and the clarity of the ratio spectra. Optimal conditions were identified during preliminary trials, ensuring reliable measurements. As ratio spectra based methodologies require several preliminary trials to optimize its selectivity and sensitivity it can be considered relatively green in comparison to more straightforward methods as FSD or ISM.

#### Ratio derivative spectrophotometric method (RDM)

Several parameters were optimized, including the divisor concentration, selection of the measurement wavelength (λ), and linearity of the resulting ratio spectra. Based on these criteria, the optimal divisors identified were TRIM at 10 µg/mL for the determination of HCTZ (Fig. 9c) and HCTZ at 4 µg/mL for the quantification of TRIM (Fig. 9d). The obtained ratio spectra were subsequently analyzed to compute the first derivative of the ratio spectra. Calibration curves were generated by plotting the amplitudes at 283 nm for HCTZ and at 251 nm for TRIM against their respective concentrations, with the regression data presented in Table 1.

### Greenness assessment of the GbD based approaches

The assessment of greenness in analytical methodologies has traditionally relied on various metrics that evaluate the development stages of these methods according to a predetermined scoring system. A persistent challenge has been the integration of greenness within the earliest stages of method development to ensure a reduced ecological footprint. Fortunately, the GbD approach addresses this challenge by employing a combined in-silico and in-vitro investigation strategy, optimizing method parameters to achieve desired analytical goals with minimal ecological impact.

The incorporation of the GbD approach in UV spectroscopic methodologies necessitated the examination of various solvent systems and their effects on the spectral bandwidths of pharmaceutical compounds in mixtures. This was accomplished through computer-based simulations^48^ including dynamic simulations and photochemical quantum approaches. These in-silico techniques allowed us to explore the relationship between solvent systems and solute molecules, enhancing our understanding of photochemical quantum phenomena, particularly the extent of peak broadening in their spectra due to solute-solvent interactions. This strategy facilitated the development of analytical methodologies that reduced analytical effort while minimizing time, energy, and chemical waste^49,50^.

To evaluate the greenness of the GbD-based methodologies, we applied two assessment protocols, including the AGREE (Analytical Greenness metric) and the Green Analytical Procedure Index (GAPI). The AGREE approach is a contemporary metric developed to quantitatively evaluate the environmental sustainability of analytical procedures. It is founded on the twelve principles of Green Analytical Chemistry (GAC)^51^ with each principle assessed individually and scored from 0 (non-green) to 1 (fully green). These principles account for factors such as solvent and reagent safety, energy use, waste production, sample throughput, and overall method efficiency^52,53^.

To calculate the AGREE score, normalized values are assigned to each of the twelve principles based on method-specific data. These are then averaged to produce a final score between 0 and 1, reflecting the overall greenness of the method. This score is visually communicated through a circular pictogram divided into 12 colored sections green for strong compliance, yellow for moderate, and red for weak with the numerical score placed at the center. This format enables a clear, at-a-glance understanding of both the strengths and limitations of a given method from a sustainability perspective.

**Fig. 10:**
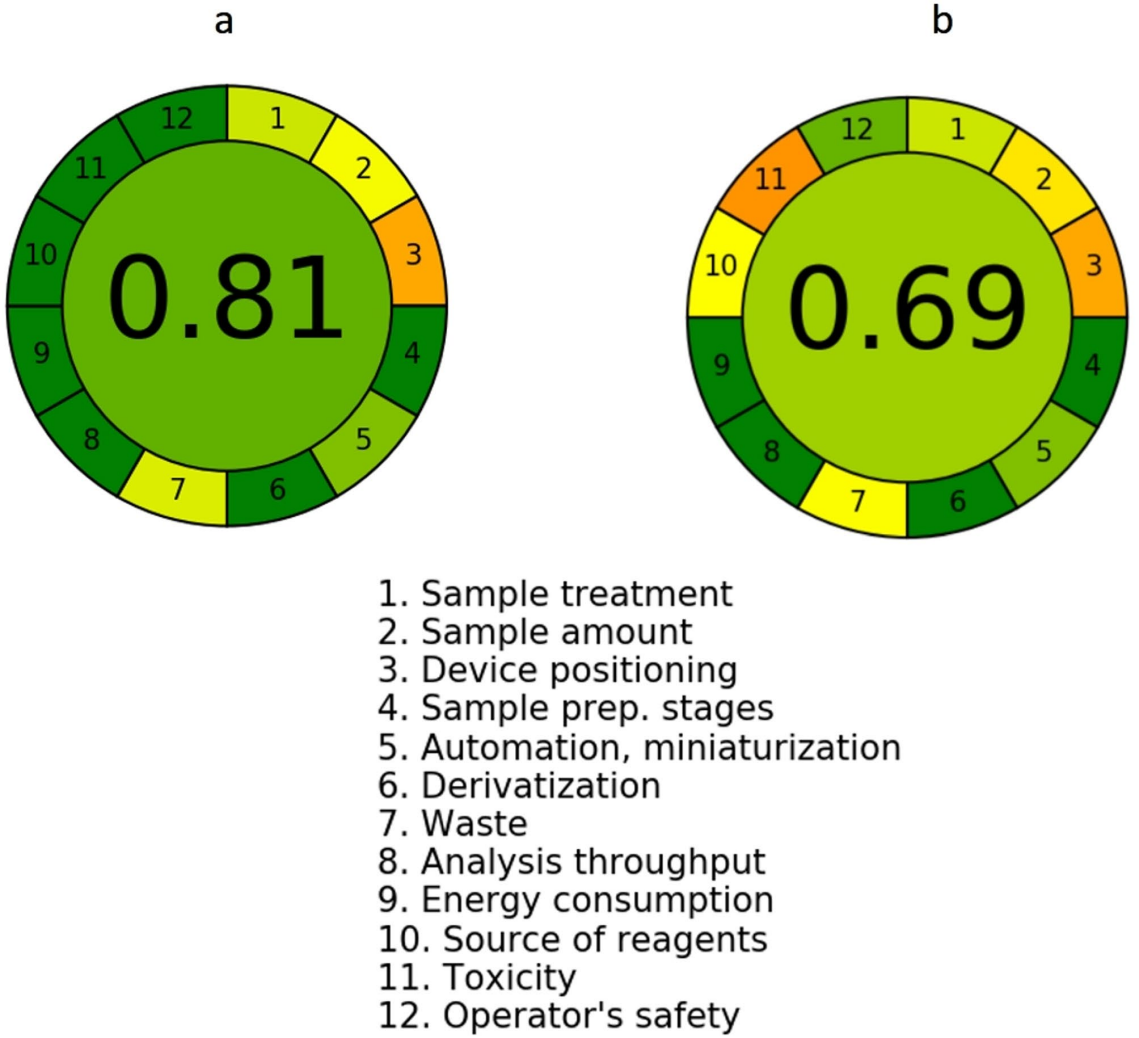
**(a)** Application of AGREE metric to assess the greenness of the proposed analytical methodologies showing appreciable score of 0.81. **(b)** Application of AGREE metric to assess the greenness of the reported analytical methodologies showing appreciable score of 0.69.

In our current work, the AGREE evaluation was performed using the official AGREE software. The developed method achieved a high score of 0.81 (Fig. 10**(a)**), confirming its strong alignment with green chemistry principles. By comparison, the previously reported method yielded a lower AGREE score of 0.69 (Fig. 10**(b)**), indicating higher environmental burden^17^. This clearly demonstrates that the proposed method is not only analytically effective but also significantly greener and more eco-friendly than the existing approach^54^.

Also, GAPI was applied which is a comprehensive visual tool used to assess the environmental sustainability of analytical procedures throughout all stages of method execution from sample collection to final analysis. Unlike other tools that offer a single numerical score, GAPI evaluates the method through a stepwise breakdown of 15 specific criteria spread across five key domains: sample handling, reagents and solvents, instrumentation, waste management, and occupational safety^55^. Each parameter is independently assessed and color-coded: green denotes low environmental impact, yellow indicates a moderate burden, and red signals a high or undesirable effect. These assessments are displayed in a hexagonal pictogram, which offers a clear visual summary of the method’s overall greenness. The dominance of green fields in the chart reflects a well-aligned method with sustainable practices, while red areas point to potential areas for environmental improvement.

To construct the GAPI profile, each component of the analytical method is carefully analyzed based on green chemistry criteria, and the corresponding colors are assigned to the relevant sections of the pictogram. Although GAPI does not produce a single final score, the visual distribution of colors offers a powerful, semi-quantitative measure of a method’s sustainability performance.

**Fig. 11:**
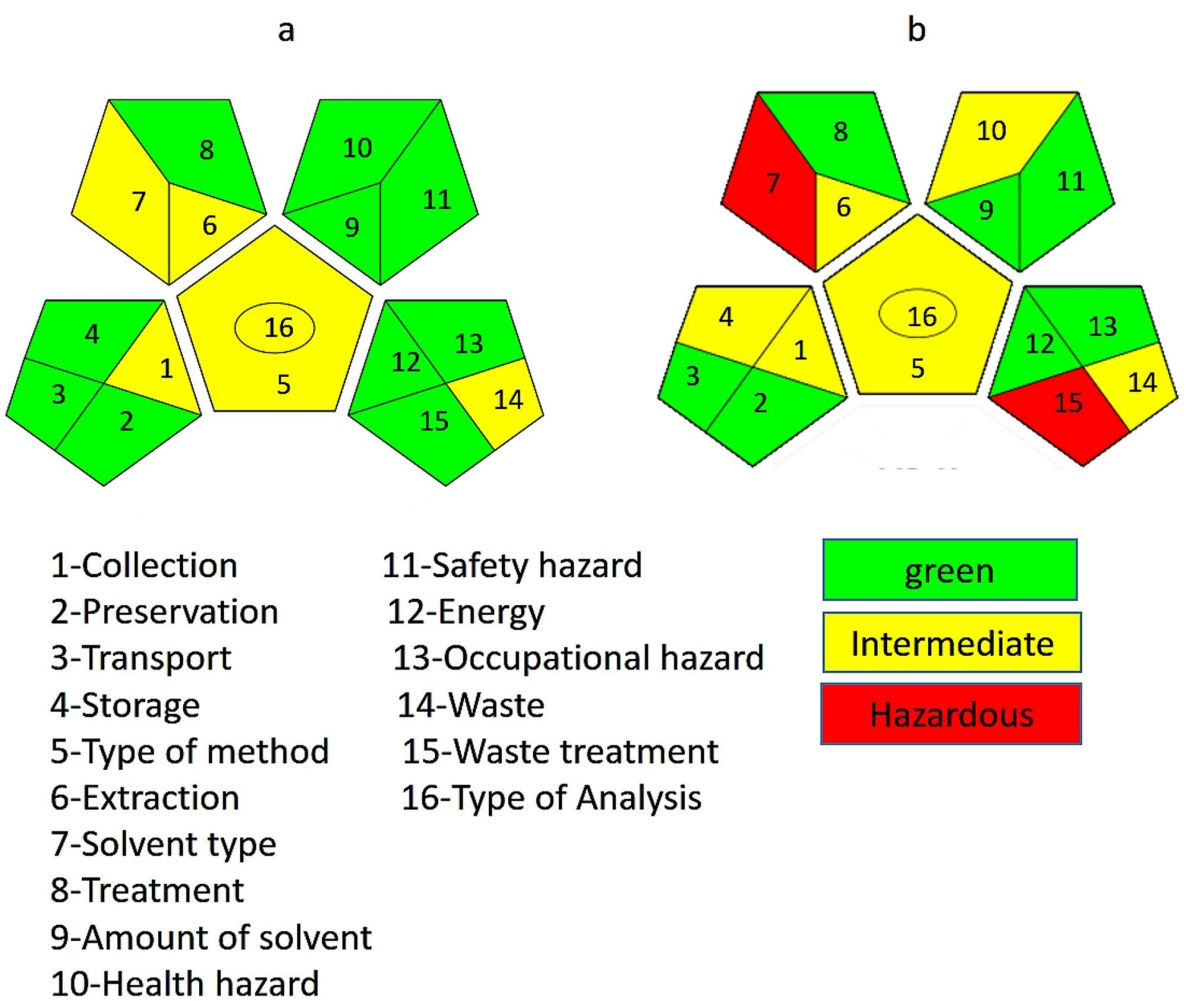
**(a)** Application of GAPI metric to assess the greenness of the proposed analytical methodologies showing appreciable greenness outcomes as shown on the color code of the pictograms. **(b) A**pplication of the GAPI metric to assess the greenness of the reported analytical methodologies reveals the summarized greenness outcomes, as indicated by the color-coded pictograms, in comparison with the proposed methodologies.

In our current study, the GAPI pictogram for the proposed methods (Fig. 11a) demonstrated a strong green profile with minimal red zones, confirming the method’s alignment with environmentally friendly practices. The use of safe solvents, reduced chemical consumption, simplified sample preparation, and low waste output all contributed to this favorable evaluation. When compared to the reported method (**(**Fig. 11b), which showed more environmental weaknesses, our proposed approach proved to be significantly more sustainable. This clearly positions the developed method as a completely green and eco-friendly alternative, offering a superior choice for modern pharmaceutical analysis in line with green chemistry objectives.

Integrating the GbD approach into the development of analytical methods significantly enhances outcomes on both the AGREE and GAPI scales. By embedding sustainability principles from the outset, researchers can prioritize environmentally friendly practices and materials, leading to greater efficiency and reduced environmental impact. This proactive strategy not only fosters innovation in analytical methodologies but also supports the advancement of sustainable practices within the field by minimizing energy consumption, reducing solvent use, and selecting safer, less toxic reagents.

### Validation

Validation of the analytical methods was conducted in accordance with the International Conference on Harmonization (ICH) Q2 (R1) guidelines^56^ encompassing key parameters such as linearity, limit of detection (LOD), limit of quantitation (LOQ), selectivity, accuracy, and precision.

#### Linearity, limit of detection (LOD), and limit of quantitation (LOQ)

The linearity of the developed spectrophotometric methods for HCTZ and TRIM was assessed by measuring concentrations within the ranges specified in Table 1. The results demonstrated robust linearity, characterized by high coefficients of determination and minimal percentage relative standard deviation (% RSD). Each concentration was analyzed in triplicate, and the corresponding regression equations were computed, as detailed in Table 1.

The LOD and LOQ were established by determining the lowest concentrations that could be reliably detected and quantified, respectively, following the ICH Q2 (R1) recommendation, as outlined in Table 1.

#### Accuracy

The accuracy of the proposed methodologies was evaluated by comparing the measured concentrations of the target compounds with their known values, expressed as mean percentage recoveries presented in Table 1. Further validation was achieved through the standard addition technique, where known quantities of HCTZ and TRIM were spiked into pre-analyzed samples at three distinct concentration levels. The results indicated no interference from excipients, as shown in Table 4.

**Table 4:** Application of standard addition technique using the proposed spectrophotometric methods:.

Drug	Pharmaceutical taken (µg/ml)	Pure added (µg/ml)	Pharmaceutical found ^a^ (µg/ml)					Recovery ^b^ (%R)				
			FSD	ISM	ACM	RDF	RDM	FSD	ISM	ACM	RDF	RDM
HCTZ	4	2	3.983	3.978	3.995	4.009	4.035	99.18	100.77	99.89	100.95	100.45
		4						98.28	101.49	98.31	99.98	99.98
		6						100.13	99.77	99.32	101.12	100.98
	Mean ± %RSD							99.19 ± 0.934	100.68 ± 0.857	99.17 ± 0.805	100.68 ± 0.615	100.47± 0.500
TRIM	6	2	6.035	5.978	5.978	6.005	6.052	101.85	101.19	101.19	100.76	100.32
		4						102.62	101.42	101.42	101.23	100.45
		6						101.89	101.23	101.23	99.92	99.45
	Mean ± %RSD							102.12 ± 0.425	101.28 ± 0.120	101.28 ± 0.120	100.64 ± 0.664	100.07 ± 0.544

^a^ average of three determinations.

^b^ average of three determination.

#### Precision

The precision of the proposed methods was assessed through both repeatability and intermediate precision, as illustrated in Table 1.

#### Repeatability

For repeatability assessment, three different concentrations of HCTZ and TRIM were analyzed in triplicate on the same day. The percentage relative standard deviation (% RSD) was subsequently calculated.

#### Intermediate precision

To evaluate intermediate precision, the same concentrations used for repeatability were analyzed three times over three consecutive days. The % RSD was calculated to assess variability between days.

#### Selectivity

The selectivity of the proposed methods was evaluated by analyzing laboratory-prepared mixtures containing varying ratios of HCTZ and TRIM. The mean recovery percentage (% R) and the standard deviation (SD) were calculated and are presented in Table 2.

### Comparative statistical assessment

The recoveries of each of the proposed methods were compared statistically against the reported method^17^ using student t-test and F-test and the results are shown in Table 3. The results reveal that the calculated student t-test and F-test values were less than their critical values indicating that there was no significant statistical difference between each of the proposed sensors and the reported method. Statistical evaluation of the results and effect sizes (Eta squared) of the assays were conducted using a one-way ANOVA test at a 95% confidence interval. The results from the ANOVA test indicated that the variability in the results can be attributed to the differences among the methods. However, the analysis revealed no statistically significant difference between the proposed methods and the reference method, as shown in Table 5.

**Table 5:** One-Way ANOVA statistical analysis with a 95% confidence interval on recovery percentage data from GbD-Based spectrophotometric methods applied to Maxzide^®^ tablets.

Source of variation	Sum of squares	Degrees of freedom	Mean square	F-value	P-value
HCTZ					
Between groups	5.99	3.00	2.00	1.16 ^a^	0.38
Within groups	13.82	8.00	1.73		
Total	19.81	11.00	***η*** ^**2 b**^	0.30	
TRIM					
Between groups	8.29	3.00	2.76	2.36 ^a^	0.15
Within groups	9.35	8.00	1.17		
Total	17.64	11.00	***η*** ^**2 b**^	0.47	

^a^ The F-critical value for HCTZ and TRIM was 4.07.

^b^ Eta squared values indicating the magnitude of variability in the results that can be attributed to the differences among the methods.

## Conclusion

Here in, we successfully demonstrated the effectiveness of the GbD approach in developing UV spectrophotometric methodologies capable of simultaneously quantifying HCTZ and TRIM in both dosage forms and laboratory prepared mixtures. The methods developed included simple mathematical techniques such as Fourier Self-Deconvolution, Absorption correction method, and Isoabsorptive point method, alongside universal ratio spectra-based approaches like ratio difference and ratio derivative methods. By assimilating in-silico techniques such as MD and TD-DFT electronic dynamics simulations with UV spectroscopy, we gained valuable insights into solute-solvent interactions at both molecular and quantum levels. The GbD framework facilitated the selection of the optimal solvent system, characterized by minimal interactions with the solute, which in turn reduced peak broadening and spectral interference. This premeditated decision not only made method development easier by requiring less analytical work, but it also improved environmental sustainability by using less energy and producing less waste. While the ratio-based approaches improved quantification’s sensitivity and specificity, the straightforward mathematical techniques used offered several benefits, such as resilience and simplicity of use. These approaches’ dependability and interchangeability were confirmed by statistical comparisons that showed no discernible differences between them. Overall, as shown by the excellent results on the GAPI and AGREE greenness scales, the use of the GbD approach in our methodology demonstrates the potential for creative and sustainable practices in analytical chemistry. This accomplishment opens the door to more effective and sustainable analytical methods. Moreover, the proposed methodologies are not only simple and sustainable but also serve as effective alternatives to chromatographic techniques for routine analytical methods in quality control laboratories, especially in limited resources regions. Their ease of use requires minimal training, and they provide enhanced occupational safety, making them particularly advantageous in these settings.

## Supplementary Information

Below is the link to the electronic supplementary material.

Supplementary Material 1

## Data Availability

The datasets generated during and/or analysed during the current study are available from the corresponding author on reasonable request.
