# Supplementary material for: Computer aided greenness by design approach for resolving and quantifying Triamterene and Hydrochlorothiazide in pharmaceutical mixtures employing multiscale dynamics simulations
Source: Sci Rep. 2025 Aug 6;15:28668. doi: 10.1038/s41598-025-13486-2 (PMC12325619; doi:10.1038/s41598-025-13486-2)
Supplement: Supplementary file 1 — Supplementary Material 1 [file 41598_2025_13486_MOESM1_ESM.docx]

**Supplementary figures:**

**
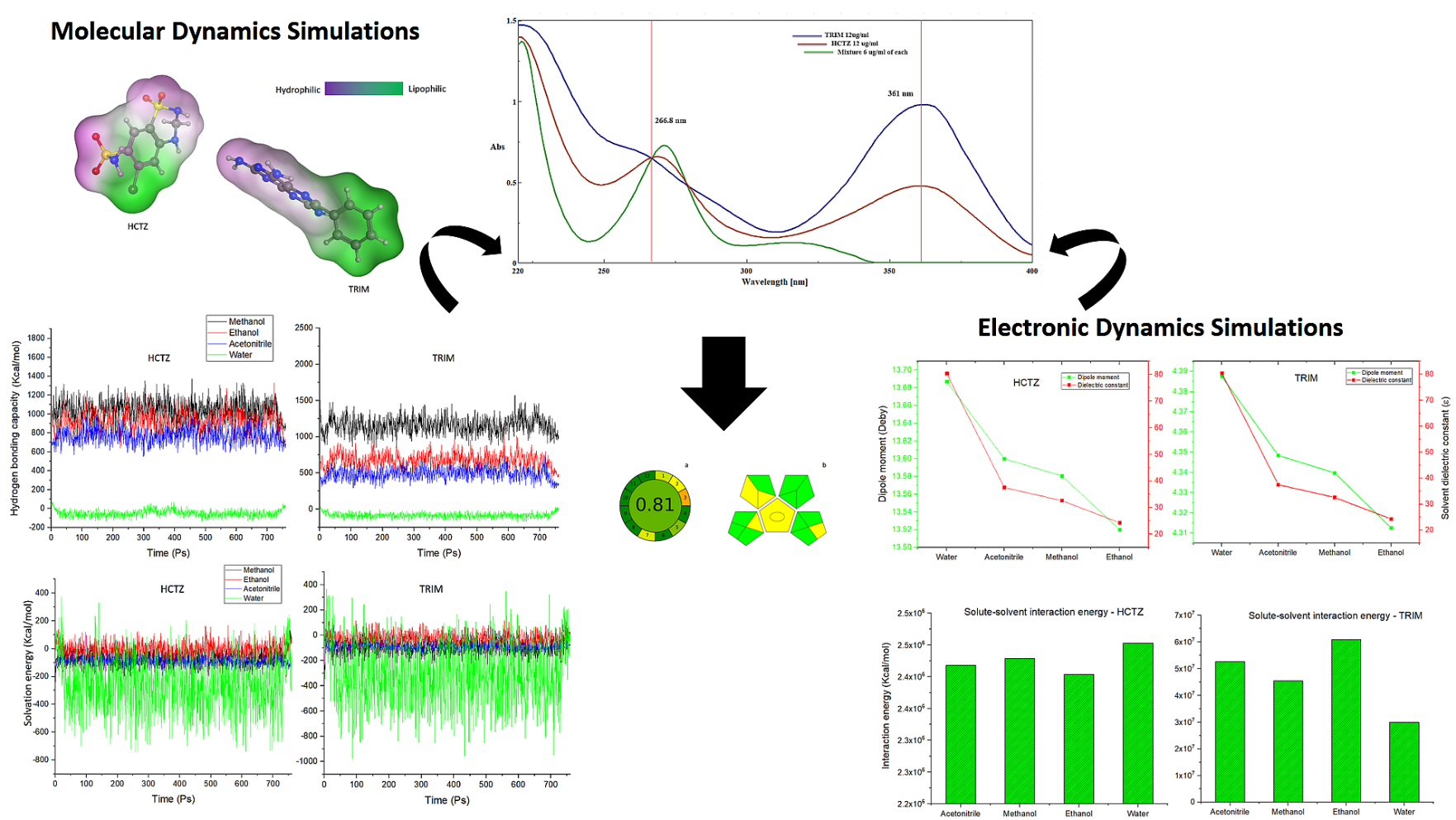
**

**Figure S1**: illustrates the general outline of the GbD approach implemented to resolve and quantify the pharmaceutical mixture of HCTZ and TRIM, highlighting its environmentally friendly outcomes. This figure was created by the authors using Microsoft PowerPoint 2010. For more information, please visit <https://www.microsoft.com/en-us/microsoft-365/powerpoint>.


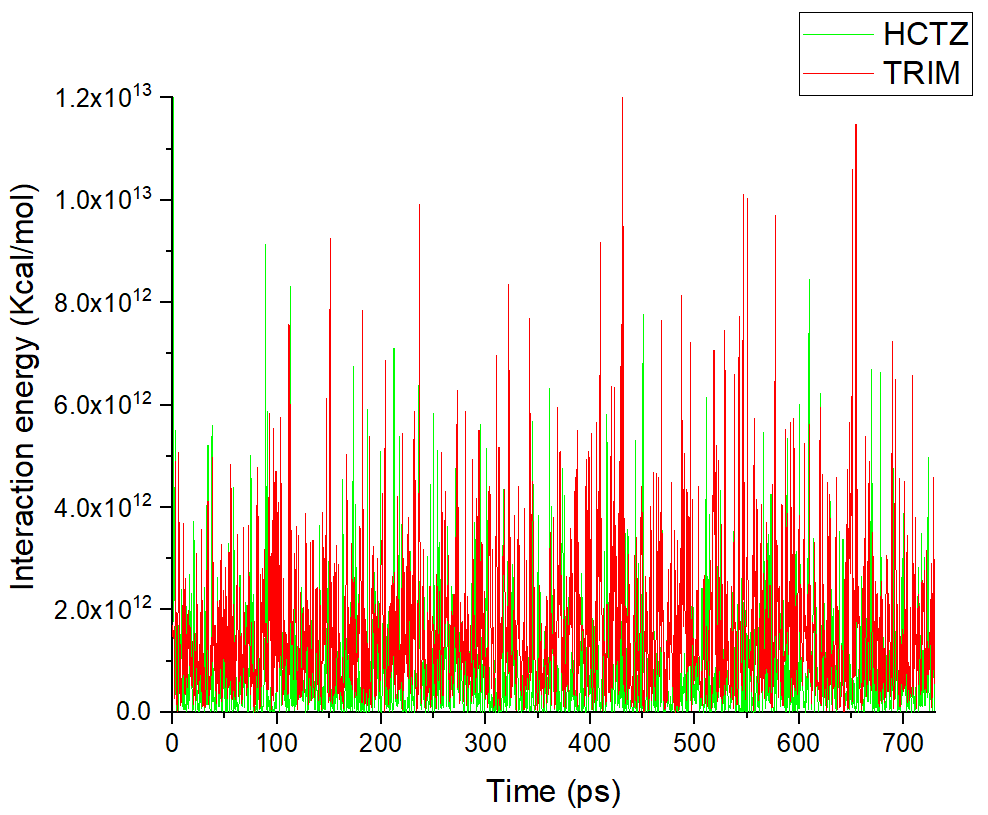


a

**Figure S2 a**: Comparison of interaction energies of HCTZ and TRIM with ethanol, illustrating the increased overall interaction of ethanol with TRIM.


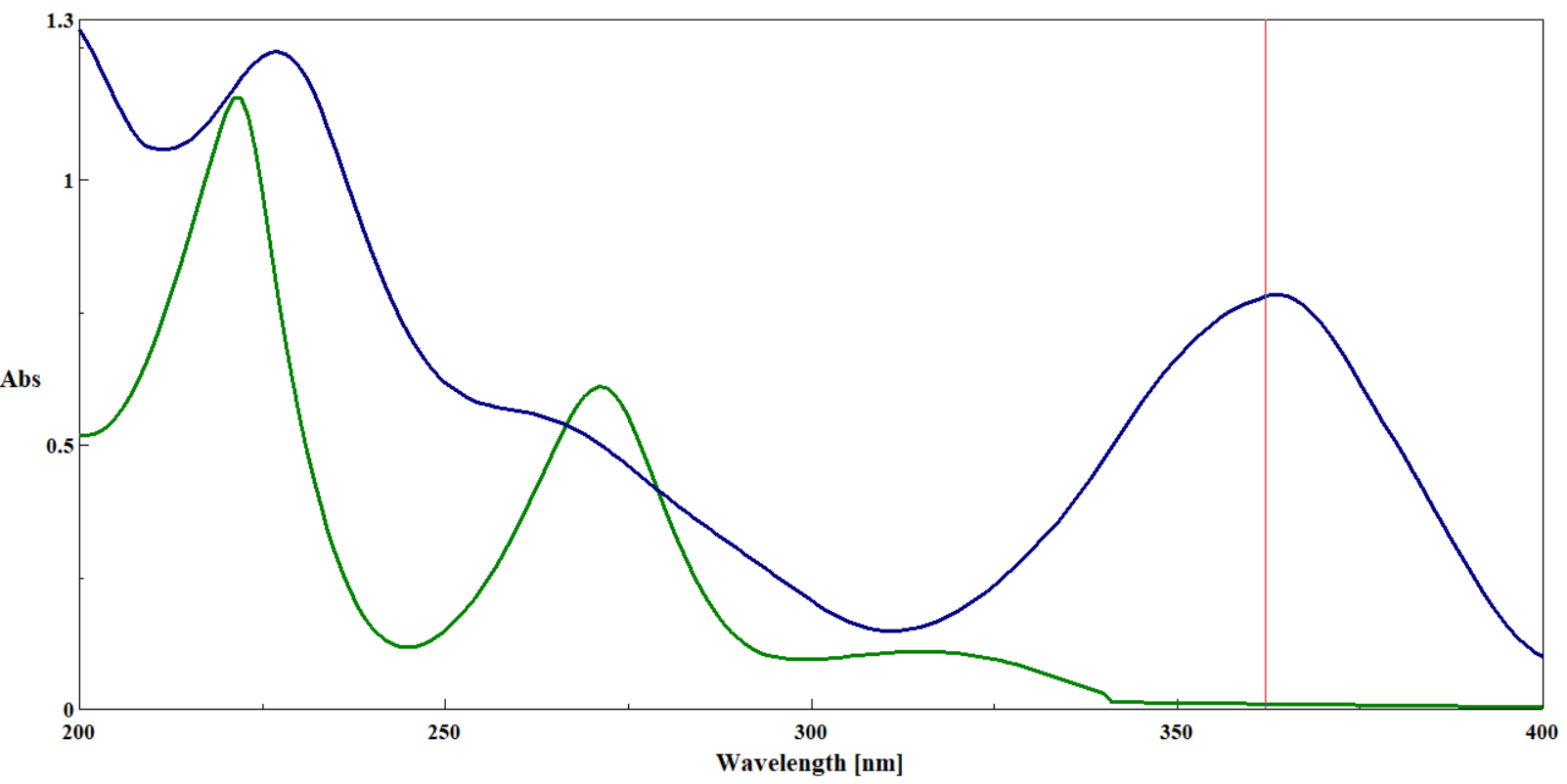


361 nm

TRIM

HCTZ

271 nm

b

**Figure S2 b**: shows the full range overlay spectra of both HCTZ and TRIM in ethanol, highlighting how ethanol broadens the TRIM peaks, particularly its n-π* transition at 361 nm.
